# Trends in the occurrence of large Whooping Crane groups during migration in the great plains, USA

**DOI:** 10.1016/j.heliyon.2020.e03549

**Published:** 2020-04-02

**Authors:** Andrew J. Caven, Matt Rabbe, Jenna Malzahn, Anne E. Lacy

**Affiliations:** a Platte River Whooping Crane Maintenance Trust, 6611 W Whooping Crane Dr., Wood River, NE, 68883, USA; b Nebraska Ecological Services Field Office, U.S. Fish and Wildlife Service, 9325 South Alda Rd., Wood River, NE, 68883, USA; c International Crane Foundation, E-11376 Shady Lane Road, Baraboo, WI, 53913, USA

**Keywords:** Flocking, Animal behavior, Landscape ecology, Whooping Crane, Migration, Wetlands, Great plains, Endangered species, Ecology, Applied ecology, Ecosystem change, Wildlife ecology, Fauna, Zoology

## Abstract

Recent detections of large gatherings of Whooping Cranes suggest that flock sizes may be increasing at some stopover locations during both the spring and fall migrations. We used the public sightings database managed by the US Fish and Wildlife Service from 1942 to 2018 to analyze data for long-term trends in group size. We then examined the spatial distribution of large groups to explore potential explanations for these occurrences. The proportion of Whooping Crane groups comprised of 2, 3, and 4–6 individuals showed no trend over time. However, observations of individuals showed a declining trend and groups of 7–9 and ≥10 showed an increasing trend. The frequency of groups observed exceeding 5 and 10 individuals were better predicted by survey year than by Whooping Crane population size suggesting that an increasing population is not the sole driver of large group occurrences. Our results indicate that large groups occur disproportionately within the 50% migration corridor, at staging areas within the first or last 20–30% of the migration path, and near conservation-managed wetlands, particularly within the southern Great Plains. Our results suggest that in addition to population growth, conspecific attraction, location within the migration corridor, and habitat loss may be contributing to large group occurrences. Further research is needed to determine the degree to which these factors influence large Whooping Crane group formation. The gathering of large numbers of Whooping Cranes in a single location presents potential tradeoffs for the species. While increasing group sizes may improve threat detection and avoidance, it comes at a cost of increased disease and mass mortality risk.

## 1 Introduction

Whooping Cranes (*Grus americana*) are federally endangered, with the last remaining self-sufficient wild population of about 500 individuals migrating between wintering grounds in Aransas National Wildlife Refuge, Texas and breeding grounds in Wood Buffalo National Park, Canada (Butler and Harrell, 2018; Pearse et al., 2018a). Whooping Cranes commonly migrate as individuals, pairs, families, or in small flocks (Armbruster, 1990; Kuyt, 1992; USFWS, 1994; Austin and Richert, 2001; Hefley et al., 2015). Armbruster (1990) and Austin and Richert (2001) observed that the most frequent sightings involved one to three Whooping Cranes, regardless of the season. Whooping cranes are territorial and non-gregarious during the breeding season and on the wintering grounds (Erickson and Derrickson, 1981, Cottam, 1996; Chavez-Ramirez, 1996). However, they exhibit more gregarious behavior during migration, occasionally gathering in flocks beyond their family groups (Erickson and Derrickson, 1981; Cottam, 1996; Walkinshaw, 1996). Records of flock sizes ranging from 6 to 20 individuals have been considered noteworthy over the last century (Bent, 1963; Kuyt, 1987, 1992; Armbruster, 1990; Walkinshaw, 1996; Austin and Richert, 2001; Sharpe et al., 2001).

Incidentally, the frequency and magnitude of large Whooping Crane group sightings appears to have increased, including a count of 76 Whooping Cranes in multiple nearby flocks at Quivira National Wildlife Refuge (NWR) in the spring of 2010 and a reported count of 151 spread across a single agricultural field in the fall of 2018 in south-central Saskatchewan, Canada (Austin and Richert, 2001; Hefley et al., 2015; USFWS, 2018; FOTWW, 2018). However, this phenomenon has not been systematically investigated in recent decades. Johnsgard and Redfield (1977) proposed that an increase in migratory flock sizes was a result of population growth, but other factors such as habitat loss or changes in behavioral patterns as the species recovers may provide alternative or additional explanations (Stahlecker, 1992; Wolf and Weissing, 2012).

The Aransas-Wood Buffalo population of Whooping Cranes (AWBP) migrates through the Great Plains via a corridor that ranges from 170 km wide in the southern Great Plains to over 400 km wide near United States-Canada border (Kuyt, 1992; Pearse et al., 2018a). The 4,000 km north-south migration, undertaken biannually and predominantly diurnally, requires significant energetic expenditure (Johnson, 1981; Kuyt, 1992; Hefley et al., 2015). Therefore stopover habitats that provide secure roosting and ample foraging opportunities throughout the migration corridor are critical to sustaining migration (National Research Council, 2005; Newton, 2006; Chavez-Ramirez and Wehtje, 2012; Niemuth et al., 2018). Stopover duration can range from one day to over one month (Faanes and Lingle, 1988; PRRIP, 2017; Jorgensen and Brown, 2017). Evidence suggests that individual Whooping Cranes and family groups do not demonstrate high stopover site fidelity, however, significant portions of the population use key stopover locations (i.e. Quivira NWR, KS; Central Platte River Valley, NE, etc.) on a near annual basis (Richert, 1999; Pearse et al., 2015).

Whooping Cranes select for and depend on diverse wetland habitats during migration (Lingle et al., 1991; Jorgensen and Dinan, 2016; Pearse et al., 2017; Niemuth et al., 2018). They generally prefer to roost and forage in shallow wetlands with relatively wide unobstructed views (Armbruster, 1990; Howlin and Nasman, 2017; Pearse et al., 2017; Farnsworth et al., 2018). Whooping Cranes use a variety of wetland types for roosting as well as foraging including palustrine habitats with various levels of herbaceous emergent vegetation, lacustrine habitats, and wide shallow rivers (Pearse et al., 2017; Niemuth et al., 2018). They also use agricultural fields and mesic grasslands to forage (Lingle et al., 1991; Jorgensen and Dinan, 2016; Niemuth et al., 2018; Baasch et al., 2019). Whooping Cranes have diverse omnivorous diets that to a large extent are derived from wetlands, which include small bodied fishes, crustaceans, herpetofauna, insects, and native wetland plants in addition to waste grains exploited particularly during migration (Allen, 1954; Blankinship, 1976; Kuyt, 1987; Bergeson et al., 2001; Geluso et al., 2013; Dinets, 2016; Caven et al., 2019a). However, the network of wetlands throughout the Great Plains has been severely degraded by development, sedimentation from adjacent agricultural landscapes, and over appropriation of water resources for human use (Cariveau et al., 2011; Horn et al., 2012; Johnson et al., 2012; Tang et al., 2012; Wright and Wimberly, 2013).

Within the Whooping Crane migration corridor wetland habitat loss has been arguably most pronounced in the playa wetlands within the southern and south-central Great Plains, south of the Platte River (Stahlecker, 1992, 1997; Cariveau et al., 2011; Tang et al., 2012). Nonetheless, there are a series of large playa wetland complexes critically important to waterbird migration in this region that have been managed for the benefit of migratory birds (USFWS, 1978; Skagen and Knopf, 1994; Aber et al., 2016). Skagen and Knopf (1994) argue that stopover site fidelity in the southern Great Plains may be higher for shorebirds at locations such as Quivira NWR, Kansas, with predictable rather than ephemeral wetland habitat availability. Cheyenne Bottoms Wildlife Area, Kansas, and Salt Plains NWR, Oklahoma, also represent some of the largest, most diverse, and most dependable wetland ecosystems in the Great Plains south of the Platte River, Nebraska (Fellows et al., 2001; Aber et al., 2016). If large Whooping Crane groups disproportionally occur in quality wetlands where appropriate habitat is otherwise regionally limited it may suggest that habitat loss is contributing to this phenomenon.

Behavioral patterns may also influence the occurrence of large Whooping Crane groups. Whooping Cranes exhibit conspecific attraction during migration and outside of traditional wintering and breeding territories (Chavez-Ramirez, 1996; Cottam, 1996). This behavior can be a signal of resource availability in ecosystems where food is unevenly distributed but temporarily plentiful (Alonso et al., 1994; Muller et al., 1997; Sundar, 2006). Flocking also allows individuals to increase their foraging rate, as there is less need for individual predator vigilance due to the collective benefit of group vigilance (Alonso et al. 1994, 2004). Additionally, conspecific attraction may represent an increasingly common behavioral pattern for Whooping Cranes as the species recovers and increasingly exhibits broader behavioral variability (Wolf and Weissing, 2012; Thompson, 2018).

We investigated the statistical trend in Whooping Crane group size across several gradients from a database of public sightings spanning over seven decades. We then explored potential factors influencing trends including population growth, habitat loss, and conspecific attraction by examining the spatial distribution of large Whooping Crane group observations. We predicted that the frequency of large group observations would increase over the last seven decades, both in terms of raw numbers and as a proportion of total observations. We hypothesized that time, on various scales (decadal, annual), would be a better predictor of Whooping Crane group size via multiple metrics than estimated population size, which would indicate that population growth is not the sole factor promoting large Whooping Crane group occurrences.

Additionally, we hypothesized that conspecific attraction was a contributing factor to large group formation (Cottam, 1996; Muller et al., 1997). Therefore, we expected large groups to occur disproportionately within the 50% migration corridor delineated by Pearse et al., 2018a, Pearse et al., 2018b, where migration is densest and Whooping Crane flocks would be most likely to encounter each other. We also posit that large Whooping Crane groups occur as a result of multiple smaller groups incidentally encountering each other where wetland habitat availability is limited (Fellows et al., 2001; Sundar, 2006). Therefore, we expect that perennial wetland sites in the south-central Great Plains, where wetland habitat is relatively limited regionally (Stahlecker, 1992; Reese and Skagen, 2017), will host a disproportionate number of large groups. We suspect that the effects of habitat loss, conspecific attraction, and population growth are additive and therefore expect to find support for all of these hypotheses to varying degrees.

## 2 Materials and methods

### 2.1 Data collection

We used a public sightings database maintained by the United States Fish and Wildlife Service (USFWS) to test for trends in the group size of AWBP Whooping Cranes migrating through the Great Plains of the United States using opportunistic observation data collected from 7 April 1942 to 1 May 2018. Collectively, members of the public, private, state and federal officials, conservation officers and professional biologists combine resources to collect and store information regarding Whooping Crane sightings during migration (Tacha et al., 2010). We use the term “group” instead of “flock” as cranes detected interacting or sharing a general roost location are categorized into one group. This methodology has been consistently applied over time and should not influence group size trends in the data (Austin and Richert, 2001). Given the nature of citizen science and observational data, only confirmed sightings were included in the analysis. Confirmed sightings are those verified in-field by a qualified individual (e.g. wildlife biologist, natural resources professional, or an “experienced observer” etc.) or based on conclusive evidence obtained by the observer (photos, descriptive details, etc.). Data associated with this manuscript can be obtained from the U.S. Fish and Wildlife Service, Nebraska Ecological Services Field Office, Wood River, Nebraska, USA, by written, electronic, or verbal request (nebraskaes@fws.gov; see “Additional information” subheading of this manuscript’s “Declarations” section for more details).

### 2.2 Data analysis

First, we calculated a number of dependent variables (n = 17; Appendix 1) that could be regressed by year to elucidate trends in group size including, the number of individual Whooping Cranes observed per group throughout the migration corridor (no. 1) and during the spring and fall migrations (nos. 2, 3), the number of juvenile Whooping Cranes per group (no. 4), and the number of adults per group (no. 5). We then calculated the maximum number of whooping cranes observed in one group per year (no. 6), spring (no. 7), and fall migration (no. 8). We also calculated the number of Whooping Crane groups larger than five and ten individuals observed per year (nos. 9, 10), spring (nos. 11, 12), and fall migration (nos. 13, 14). Finally, we calculated the mean number of Whooping Cranes observed per group for each survey year (no. 15), spring (no. 16), and fall migration (no. 17). We summarized univariate statistics for these dependent variables including the minimum, maximum, lower quartile (25^th^ percentile), upper quartile (75^th^ percentile), mean, and median values.

All analyses were conducted using the statistical software program R 3.2.1 (R Core Team, 2015). We used the “MASS” package to analyze data using generalized linear models (Venables and Ripley, 2002). We used the “distplot” function in the “vcd” package to identify the best distributional fit for the dependent variables in our models (Meyer et al., 2017). Our data best fit a Poisson distribution (1–14) in all cases except those evaluating the mean group size per year, spring, or fall (15–17), which best fit a “Gaussian” distribution. All models were bivariate, regressing dependent variables assessing group size by year or Whooping Crane population size (Hurvich and Tsai, 1989; Butler and Harrell, 2018). Year and Whooping Crane population size were highly correlated (*r* = 0.91, *p* < 0.001) and therefore could not be included as independent variables in the same model. However, we were able to compare their model fit in explaining various dependent variables using McFadden's Pseudo *R*^2^ statistic (McFadden, 1974). We utilized the “pscl” package to calculate McFadden's pseudo *R*^2^ (MF *R*^2^; McFadden, 1974; Jackman et al., 2017). MF *R*^2^ evaluates the fit of a model in relation to the null model (as opposed to estimating the amount of variation in the dependent variable accounted for) and values of 0.2–0.4 are generally indicative of a good model fit, and values of >0.4 indicate an exceptional model fit (Smith and McKenna, 2013; Negri, 2016).

We examined the percentage of total groups observed of various size classes (1, 2, 3, 4–6, 7–9, & ≥ 10) per year and decade to determine if the proportion of Whooping Cranes detected in large groups has increased over time. We used least squares regression models including linear, exponential (log-transformed dependent variable), and non-linear power models to fit curve lines associated with trends in the percent of observations comprised of various group sizes by year and decade using the “stats” package (R Core Team, 2015; Rossiter, 2016). We assessed the fit of various curves lines to trend data using a variety of diagnostics including data and residual plots (distribution, Cook's distance, etc.), adjusted *R*^2^ values, and Akaike Information Criterion corrected for small sample sizes (AICc) using the “MuMIn”, “gglot2”, and “graphics” packages (Wickham, 2009; Burnham et al., 2011; R Core Team, 2015; Barton, 2016; Rossiter, 2016). We report AICc weights as the primary model fit statistic for non-linear power models (comparing them to log-transformed, linear, and null models of the same bivariate relationship) and adjusted *R*^2^ values for linear and exponential models in the results (Xiao et al., 2011; Rossiter, 2016). Decadal analyses (n = 7 decades) included 1942 to 1959 as one observation period, though it exceeded a decade in length, to ensure a sufficient sample size to accurately represent the proportional occurrence of various group sizes as total observations were limited during this period (n = 26). The most recent observation period also did not span exactly one decade and included 9 years of data (2010–2018; n = 1,043 observations). We dropped all years with less than 5 total Whooping Crane group observations regarding annual trend analyses to prevent yearly proportional distributions from being highly skewed (n = 59; 10 years dropped).

We compared Whooping Crane group size across states within the 95% migration corridor (Pearse et al., 2018a) using a one-way ANOVA with a Tukey Honest Significant Differences (HSD) post-hoc test to determine if group size differed across space (“stats package”). Though states are socio-political boundaries, those in the central Great Plains are clearly ordered along a north-south axis and aside from Texas and Montana, the states included in this analysis are all relatively similar in size and shape and therefore represent relatively discrete units of the central flyway (OK: 181,196 km^2^/69,960 mi^2^, KS: 213,099 km^2^/82,278 mi^2^, NE: 200,356 km^2^/77,358 mi^2^, SD: 199,730 km^2^/77,116 mi^2^, ND: 183,273 km^2^/70,762 mi^2^). Austin and Richert (2001) also used state boundaries to examine Whooping Crane stopover data in their comprehensive analysis of public sightings data. Also, state boundaries can relate to important differences in policy that impact wetland health (Dallimer and Strange, 2015). We also calculated mean group size and tallied the number of group observations by state for both spring and fall migrations to examine differences therein. We used bivariate Poisson regression models to examine the relationship between latitude and group size throughout the migration corridor, during the spring and fall migration periods, and to analyze trends in group size by state (“MASS” package, R.3.2.1, Venables and Ripley, 2002).

We manually vetted the locations of Whooping Crane groups exceeding ten individuals (>95^th^ percentile) and 15 individuals (>98^th^ percentile) to best summarize where the largest aggregations of Whooping Cranes occurred geographically using both Google Earth Pro Version 7.3 (Google, 2018) and ArcGIS 10.5.1 (ESRI, 2016). We recorded the names of conservation properties, their ownership, major water bodies used, and critical habitat statuses where applicable, and provide a brief summary of key locations. We documented if Whooping Crane groups were located on private or conservation lands (federal, state, or non-governmental conservation organization ownership), in addition to if groups were using areas designated critical habitat (USFWS, 1978). We also recorded if critical habitat or other conservation lands existed within 15 km of Whooping Crane groups as areas within this range can be reached within daily movements during stopover events (Pearse et al., 2017). Using the polygons delineating the 50% use area of the Whooping Crane migration corridor developed by Pearse et al., 2018a, Pearse et al., 2018b, we calculated the percentage of flocks of 10 or more occurring within the 50% core migration area (including the confidence intervals surrounding the 50% corridor delineation).

## 3 Results

**Figure 1:**
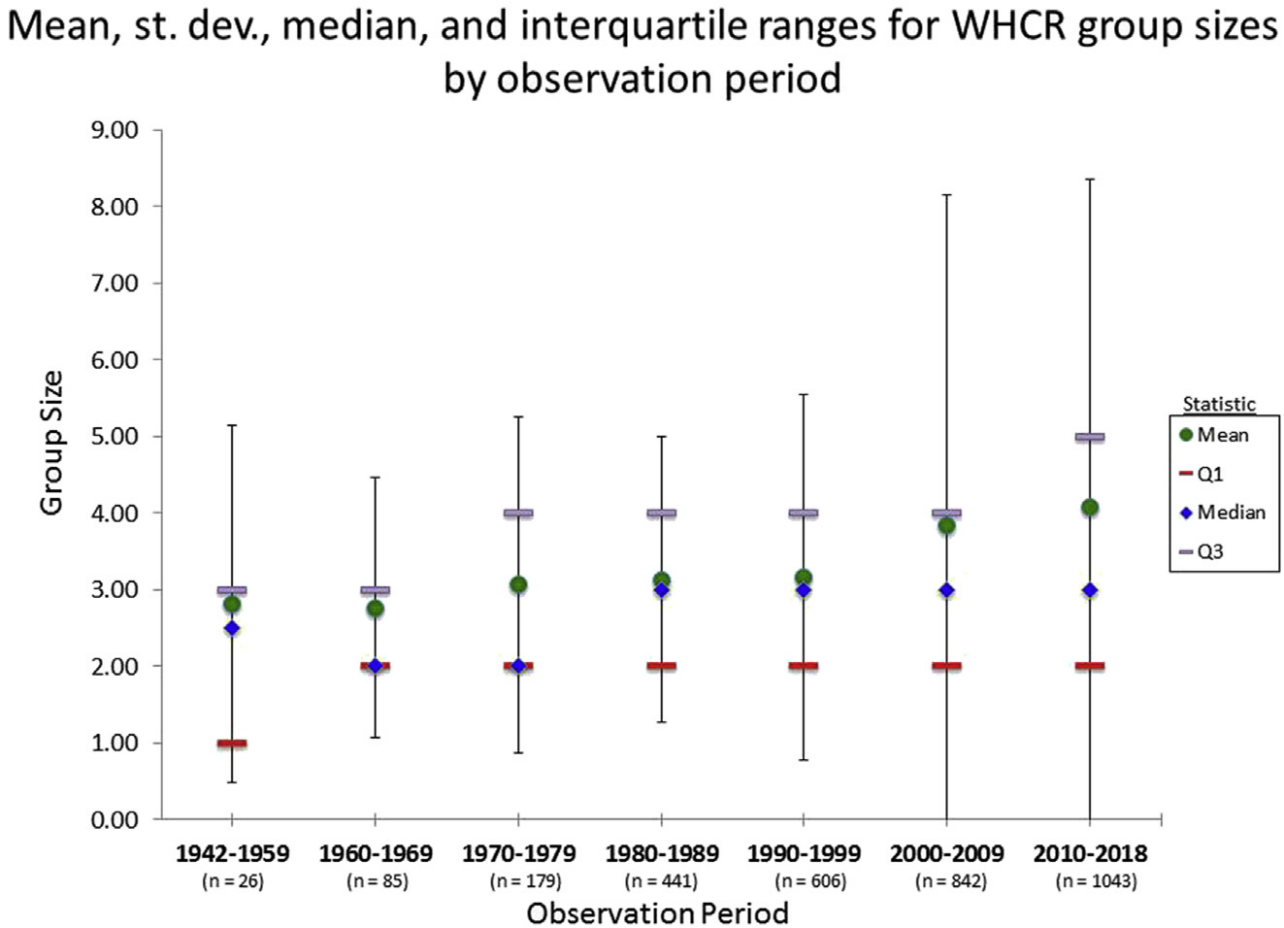
Summary statistics regarding Whooping Crane “group size” by decadal “observation period” including mean (green circle), median (blue diamond), interquartile ranges (Q1 = 25^th^ percentile, red bar; Q3 = 75^th^ percentile, gray bar), and standard deviation (1 SD) confidence intervals confidence intervals surrounding mean (black lines). Observation periods all include 10 years with the exception of the first (1942–1959) and last (2010–2018).

The mean number of Whooping Cranes per group was 3.6 individuals with a maximum of 76 observed in a single group (Table 1). The mean maximum group size observed per survey year was 12.1, with a mean of 9.3 and 9.9 for the spring and fall respectively (Table 1). Mean group size observed per year ranged from 1.0 to 8.0 individuals, with both the minimum and maximum values observed in the first decade of the study when there were far fewer sightings logged per year and any observation would highly impact mean values (Table 1). The mean count of groups exceeding 5 individuals observed per year was 10.0 (max. = 49), with the fall (*X̄* = 6.3, max. = 39) generally having more groups of 5 or more individuals than the spring (*X̄* = 4.1, max. = 23). On average there were just over two groups exceeding 10 individuals observed per year with a maximum of 22 in 2008 (Table 1). Median group size ranged between 2.0 and 2.5 from 1942 to 1979, and has consistently been 3.0 across the last 4 decades (1980–2018; Figure 1). Mean group size slowly increased from about 2.8 in 1942–1959 to 3.2 in 1990–1999, and has increase more sharply over the last two decades to 3.8 in 2000–2009 and 4.1 in 2010–2018 (Figure 1). The lower quartile value was 1.0 in 1942–1959 and has remained consistent at 2.0 Whooping Cranes across the last six decades (Figure 1). However, the upper quartile has steadily increased, ranging from 3.0 (very near the mean) in 1942–1959 to 5.0 in 2010–2018 (Figure 1). The deviation in group size has also increased significantly, particularly within the last two decades, despite a consistently increasing sample size across decades (Figure 1).

**Table 1:** Summary statistics including minimum (Min.), lower quartile (Q1; 25^th^ percentile), Median, Mean, upper quartile (Q3; 75^th^ percentile), and maximum (Max.) values for dependent variables used to assess the trends in the size of migrating groups of AWBP Whooping Cranes from 1942 to 2018.

Dependent Variable	Min.	Q1	Median	Mean	Q3	Max.
No. per Group	1	2	3	3.6	4	76
No. per Group – Spr.	1	2	3	3.6	4	76
No. per Group – Fall	1	2	3	3.7	4	36
Juveniles per Group	0	0	0	0.4	1	11
Adults per Group	0	2	2	3.3	4	65
Max in Group per Yr.	1	6	9	12.1	13	76
Max in Group per Spr.	0	5	7	9.3	11	76
Max in Group per Fall	0	4.8	8	9.9	12.3	36
Mean in Group – Yr.	1.0	2.7	3.2	3.3	3.8	8.0
Mean in Group – Spr.	0.0	2.6	3.0	3.1	3.6	8.0
Mean in Group – Fall	0.0	2.7	3.0	3.0	3.6	6.7
Count of Groups >5 – Yr.	0	1	6.5	10.0	14	49
Count of Groups >5 – Spr.	0	1	2	4.1	6	23
Count of Groups >5 – Fall	0	0.8	4	6.3	8.5	39
Count of Groups >10 – Yr.	0	0	0	2.3	2	22
Count of Groups >10 – Spr.	0	0	0	0.8	1	10
Count of Groups >10 – Fall	0	0	0	1.5	1.3	21

**Figure 2:**
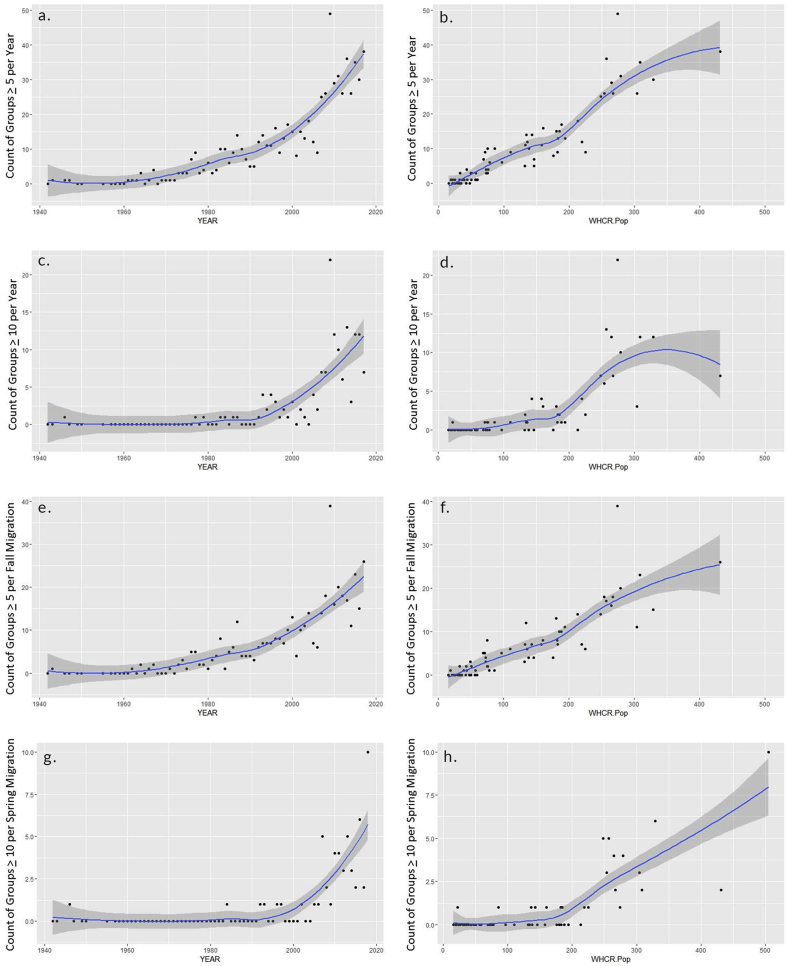
Count of Whooping Crane groups of five or more and ten or more observed per annum or migration season (spring, fall) by year (a, c, e, g; n = 1942–2018) or by Whooping Crane population (b, d, f, h; n = 16–505).

We detected a statistically significant increasing annual trend in migratory group size from 1942 to 2018 via all metrics investigated (17 models) including number per group, juveniles per group, adults per group, maximum group size, count of groups of 5 or more as well as 10 or more individuals, and mean group size (Table 2). We also detected a significant relationship between Whooping Crane population and several metrics including max group size, count of groups of 5 or more as well as 10 or more individuals (9 total models; Table 2). We ran a total of 26 models assessing some metric of Whooping Crane group size by either observation year or Whooping Crane population size, with 13 of those models exhibiting excellent model fit with MF *R*^2^ values of between 0.41 and 0.67. The eight models that had the best data fit (MF *R*^2^ >0.50) were all associated with counts of groups exceeding 5 or 10 individuals per annum or season by observation year (6 models) or Whooping Crane population size (2 models) (Table 2, Figure 2). Models with observation year as the predictor variable outperformed corresponding models with Whooping Crane population as the predictor in all cases. For instance, the count of Whooping Crane groups of 5 or larger per annum was better predicted by survey year (MF *R*^2^ = 0.67) than by Whooping Crane population (MF *R*^2^ = 0.57; Table 2, Figure 2). Similarly, the count of Whooping Cranes groups of 10 or larger by fall migration similarly demonstrated a better model fit with observation year (MF *R*^2^ = 0.51) than Whooping Crane population (MF *R*^2^ = 0.41; Table 2). The top three models of any combination of independent and dependent variables ranked by MF *R*^2^ were: i) count of groups exceeding 5 individuals by year, ii) count of groups exceeding 5 individuals per fall, and iii) count of groups exceeding 10 individuals per year (Table 2, Figure 2).

**Table 2:** Bivariate generalized linear models assessing trends in the group size of AWBP Whooping Cranes migrating through the central Great Plains of the United States by survey year (“Year” = 1942–2018) or Whooping Crane population (“WHCR *N*” = 16–505).

IV	DV	Data	*Β*	SE	*z*	*p*	Pseudo*-R*^*2*^	df
Year	No. per Group	All	0.0094	0.0007	13.41	<0.001∗∗∗	0.011	3220
Year	No. per Group	Spr.	0.0074	0.0011	6.89	<0.001∗∗∗	0.008	1258
Year	No. per Group	Fall	0.0108	0.0009	11.56	<0.001∗∗∗	0.014	1947
Year	Juv. per Group	All	0.0083	0.0022	3.77	<0.001∗∗∗	0.003	3220
Year	Adults per Group	All	0.0096	0.0007	12.92	<0.001∗∗∗	0.011	3220
Year	Max in Group	Yr.	0.0304	0.0019	15.95	<0.001∗∗∗	0.379	67
Year	Max in Group	Spr.	0.0313	0.0022	14.29	<0.001∗∗∗	0.322	67
Year	Max in Group	Fall	0.0339	0.0022	15.38	<0.001∗∗∗	0.414^+^	66
WHCR *N*	Max in Group	Yr.	0.0044	0.0003	17.14	<0.001∗∗∗	0.331	67
WHCR *N*	Max in Group	Spr.	0.0045	0.0002	15.69	<0.001∗∗∗	0.291	67
WHCR *N*	Max in Group	Fall	0.0054	0.0003	16.11	<0.001∗∗∗	0.357	66
Year	Count of Groups >5	Yr.	0.0596	0.0027	21.82	<0.001∗∗∗	0.674^**+**^	66
Year	Count of Groups >5	Spr.	0.0592	0.0042	14.05	<0.001∗∗∗	0.524^+^	67
Year	Count of Groups >5	Fall	0.0611	0.0035	17.40	<0.001∗∗∗	0.599^**+**^	66
WHCR *N*	Count of Groups >5	Yr.	0.0081	0.0003	25.0	<0.001∗∗∗	0.569^+^	66
WHCR *N*	Count of Groups >5	Spr.	0.0067	0.0004	16.8	<0.001∗∗∗	0.436^+^	67
WHCR *N*	Count of Groups >5	Fall	0.0082	0.0004	20.1	<0.001∗∗∗	0.507^+^	66
Year	Count of Groups >10	Yr.	0.0984	0.0084	11.76	<0.001∗∗∗	0.590^**+**^	66
Year	Count of Groups >10	Spr.	0.1144	0.0155	7.37	<0.001∗∗∗	0.540^+^	67
Year	Count of Groups >10	Fall	0.0958	0.0099	9.72	<0.001∗∗∗	0.508^+^	66
WHCR *N*	Count of Groups >10	Yr.	0.0106	0.0007	15.22	<0.001∗∗∗	0.482^+^	66
WHCR *N*	Count of Groups >10	Spr.	0.0094	0.0009	10.85	<0.001∗∗∗	0.469^+^	67
WHCR *N*	Count of Groups >10	Fall	0.0104	0.0008	12.46	<0.001∗∗∗	0.414^+^	66
Year	Mean in Group	Yr.	0.0153	0.0054	2.85	0.0059∗∗	0.041	67
Year	Mean in Group	Spr.	0.0182	0.0066	2.75	0.0077∗∗	0.033	67
Year	Mean in Group	Fall	0.0403	0.0050	7.98	<0.001∗∗∗	0.213	66

Note: All models are Poisson regression models except for “Mean in Group” models, which have a “Gaussian” distribution. All models with a very good model fit (McFadden's Pseudo *R*^2^ > 0.4) are marked with a “+” superscript. Footnote: *p* < 0.001∗∗∗, 0.01∗∗.

**Figure 3:**
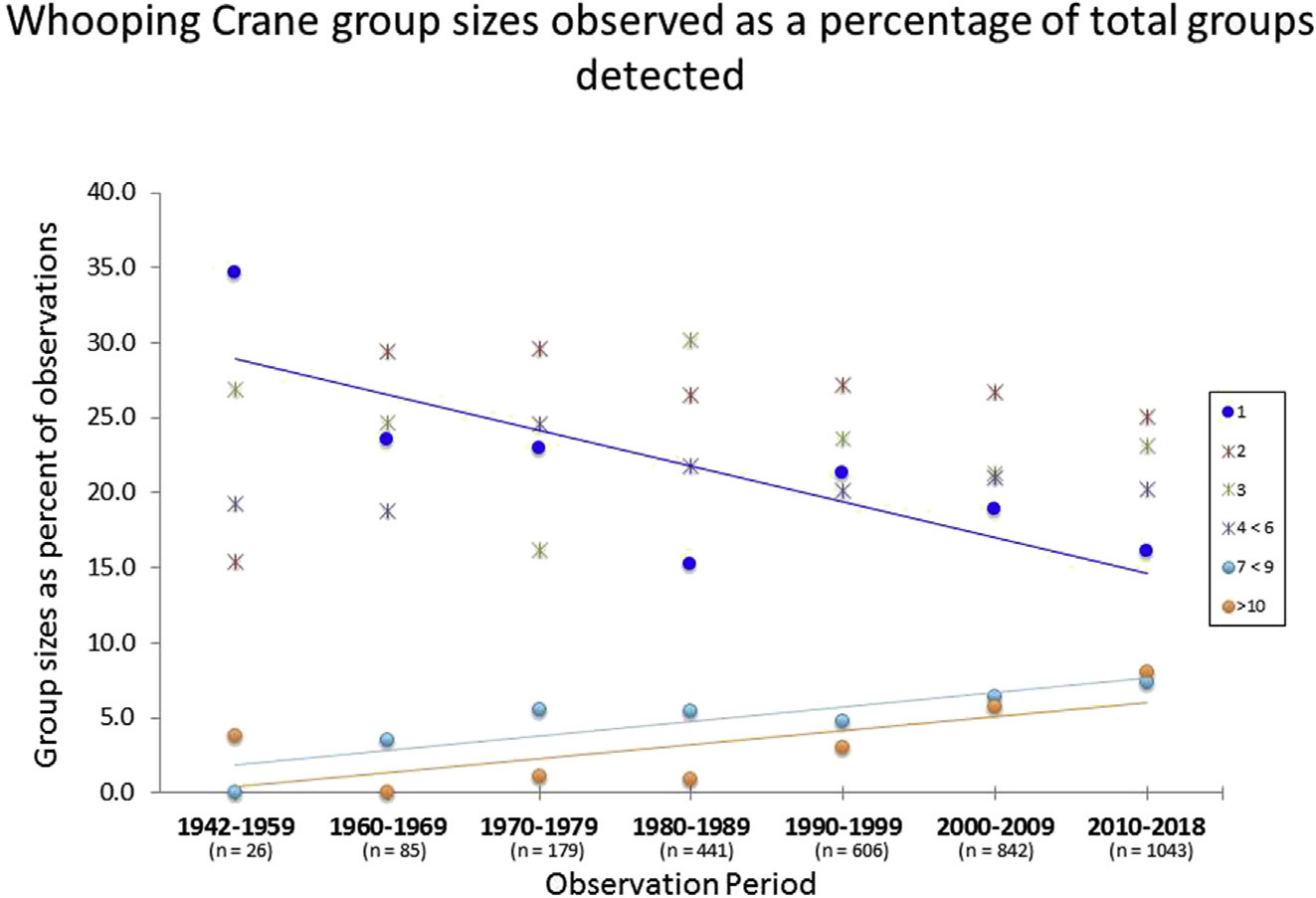
Whooping Crane group sizes observed as a percentage of total groups detected on a decadal time scale. Group sizes exhibiting a significant trend in proportional occurrence are fit with least squares linear regression lines (navy blue line = individual Whooping Cranes, sky blue line = groups of seven to nine, and orange = groups of 10 or more).

**Figure 4:**
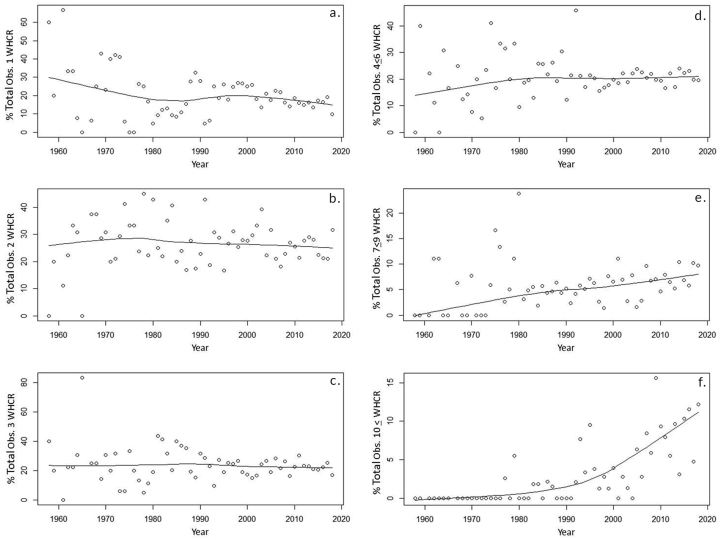
Scatterplots fit with LOESS (locally weighted smoothing) trend lines demonstrating the percentage of total Whooping Crane groups observed of various size classes by observation year from 1958-2018, including groups composed of individual (a), 2 (b), 3 (c), 4–6 (d), 7–9 (e), and ≥10 Whooping Cranes.

Groups of 2, 3, and 4–6 showed no proportional trend over an annual or decadal timescale and constituted 25.7 ± 6.5%, 23.7 ± 4.4%, and 20.8 ± 1.9% of all Whooping Crane observations respectively across decades (*X̄*±SD, n = 7; Figure 3). However, observations of individual Whooping Cranes showed a declining trend, and observations of groups of 7–9 and ≥10 showed an increasing trend at both the annual and decadal scales (Figures 3 and 4). The proportion of individual Whooping Cranes observed in relation to all other group sizes decreased -10.55 ± 3.6% in value each decade across a logarithmic curve, equating to an absolute decrease of -3.65 ± 1.25% of total observations recorded per decade based on maximum values and -1.60 ± 0.77% based on minimum values (*p =* 0.04, adj*. R* ^2^ = 0.54, log-transformed model; Figure 3). On an annual scale the percentage of groups comprised of an individual Whooping Crane decreased about -0.21 ± 0.10% per year (*p* = 0.03, adj*. R* ^2^ = 0.06, linear model; Figure 4). The percentage of groups composed of seven to nine Whooping Cranes increased by about 1.06 ± 0.06% per decade on average with the curve increasing more sharply from the late 1950s to the late 1980s than from the early 1990s until 2018 (*p <* 0.001, AICc weight = 0.93, power model; Figures 3 and 4). On an annual scale Whooping Crane groups of seven to nine individuals increased about 11.65 ± 2.08% in value each year across a logarithmic curve, equating to an absolute increase of 0.33 ± 0.06% of total observations per year based of median values and 0.89 ± 0.17% based on maximum values (*p* < 0.001, adj*. R* ^2^ = 0.32, log-transformed model; Figure 4). Whooping Crane groups of 10 or larger increased as a percentage of total groups observed by an average of 0.92 ± 0.12% per decade, with the rate of increase being higher from the early 1990s to 2018 than before (*p <* 0.001, AICc weight = 0.86, power model; Figures 2, 3, and 4). On an annual scale the percentage of groups comprised of 10 or more Whooping Cranes increased 0.16 ± 0.02% per year (*p* < 0.001, adj*. R* ^2^ = 0.50, linear model; Figure 4).

The largest number of Whooping Crane groups was observed in Nebraska (n = 491) and North Dakota (n = 303) in the spring (Table 3). However, the most groups of 10 or more individuals were observed in Kansas (n = 22) and Nebraska (n = 14) in the spring (Table 3). In the fall the highest number of Whooping Crane groups was observed in Kansas (n = 572) and North Dakota (n = 409), while the largest number of groups of 10 or more individuals was observed in Oklahoma (n = 35) and Kansas (n = 34; Table 3). Mean group size was largest in Oklahoma (spring: *X̄* = 5.45, fall: *X̄* = 4.69) and Kansas (spring: *X̄* = 4.74, fall: *X̄* = 3.92) throughout both migrations (Table 3). Comparing mean stopover group sizes between states within the 95% migration corridor in the spring, Kanas and Oklahoma did not differ significantly, but were both greater than North Dakota, South Dakota, and Nebraska, but not significantly different than Texas and Montana (Appendix 2). In the fall Oklahoma had higher mean group sizes observed than North Dakota, South Dakota, Nebraska, and Texas, but Kansas was only significantly larger than Texas. In all cases Oklahoma and Kansas were larger than Texas and Montana, but confidence intervals were larger and differences therefore insignificant likely due to smaller samples in Texas and Montana.

**Table 3:** Mean (*x̅*), standard deviation (SD), total number (n) of Whooping Crane groups, and the total number of groups of 10 or more individuals (G10+) observed in the spring and fall for states within the 95% migration corridor (See Pearse et al., 2018a).

State	Spring				Fall			
	*x̅*	SD	n	G10+	*x̅*	SD	n	G10+
TEXAS	4.03	3.04	34	2	2.63	2.41	134	2
OKLAHOMA	5.45^***1***^	5.87	58	6	4.69^***1***^	5.64	331	35^1^
KANSAS	4.74^***2***^	6.17	199	22^***1***^	3.92^***2***^	3.35	572^***1***^	34^2^
NEBRASKA	3.24	2.42	491^***1***^	14^***2***^	3.42	2.72	304	8
SOUTH DAKOTA	3.29	2.44	140	4	3.26	2.50	168	7
NORTH DAKOTA	3.12	2.34	303^***2***^	8	3.19	2.92	409^***2***^	16
MONTANA	2.91	1.81	23	0	2.82	1.81	17	0

Values for *x̅*, n, and G10+ are ranked by superscript as ^1^highest and ^2^second highest.

Group size decreased as latitude increased within the Great Plains of the United States during both the spring and fall migrations, suggesting groups decreased in size as they migrated north in the spring and increased as they migrated south in the fall (Table 4). Though statistically significant, these models did not have a good data fit (*MF R*^2^ = 0.004–0.011; Table 4), suggesting that although a real trend in group size by latitude exists within the data, latitude is not an important determinate of group size. All states with the exception of Montana demonstrated a statistically significant increase in group size from 1942 to 2018, but the model fit was poor in all cases with the best model fit exhibiting a MF *R*^2^ of 0.03 in regard to Oklahoma (Table 4).

**Table 4:** Bivariate Poisson regression models used to assess trends in the size of AWBP Whooping Crane groups migrating through the central Great Plains of the United States and select states within by latitude and year.

DV	IV	Data	*Β*	SE	*z*	*p*	Pseudo*-R*^*2*^	df
No. per Group	Latitude	All	-0.0154	0.0018	-8.51	<0.0001∗∗∗	0.004	3220
No. per Group	Latitude	Spr.	-0.0243	0.0028	-8.70	<0.0001∗∗∗	0.011	1258
No. per Group	Latitude	Fall	-0.0105	0.0023	-4.47	<0.0001∗∗∗	0.002	1947
No. per Group	Year	TX	0.0097	0.0042	2.31	0.0287∗	0.007	171
No. per Group	Year	OK	0.0159	0.0019	8.33	<0.0001∗∗∗	0.026	390
No. per Group	Year	KS	0.0115	0.0014	7.98	<0.0001∗∗∗	0.016	769
No. per Group	Year	NE	0.0066	0.0015	4.48	<0.0001∗∗∗	0.006	796
No. per Group	Year	SD	0.0088	0.0022	4.04	<0.0001∗∗∗	0.013	306
No. per Group	Year	ND	0.0071	0.0015	4.77	<0.0001∗∗∗	0.007	713
No. per Group	Year	MT	-0.0114	0.0061	-1.88	0.0603	0.024	38

Footnote: *p* < 0.001∗∗∗, 0.05∗.

Of habitats hosting groups of 10 or more Whooping Cranes, 50.6% were under some sort of conservation ownership, including 38.6% by the USFWS and 7.0% by various state wildlife/natural resource management agencies (Table 5). Major sites included Quivira NWR, and Salt Plains NWR, which respectively hosted 21.5% and 15.2% of all observations of Whooping Crane flocks 10 or larger and each supported 20.8 % of groups of 15 individuals or greater. Over 70% of sites hosting Whooping Crane groups of 10 or more were within 15 km of land managed by conservation organizations. Of habitats hosting flocks of 15 or more Whooping Cranes 56.6% were on conservation lands while 81.1% were within 15 km of them. Aside from Quivira NWR, Salt Plains NWR, Cheyenne Bottoms Wildlife Area, and the central Platte River, no site had more than 2.5% of the observations of large Whooping Crane flocks (Table 5). In all, 39.8% of flocks 15 or larger were observed within 15 km of Salt Plains NWR (Table 5). Notably, 2.5% of the groups 10 or larger were observed within the Middle Loup River, Nebraska; taken together with the main stem Loup River (1.3%), and the North Loup River (0.6%), 4.4% of groups 10 or larger were observed within the Loup River system. Though no particular prairie potholes wetland complex was utilized multiple times by groups of 10 or more Whooping Cranes, 10.1% of such groups were observed in private and public wetlands scattered throughout the prairie potholes region of North Dakota. We found that 69.0% of flocks of 10 or more individuals were documented within the 50% use area of the migration corridor delineated by Pearse et al. (2018a, b).

**Table 5:** Percent of total observations of Whooping Crane groups 10 or larger and 15 or larger by property ownership, location, and critical habitat designation as well as whether locations were within 15 km of a conservation property, critical habitat, or a key stopover site.

Land Status	>10 WHCR	>10 WHCR (≤15 km)	>15 WHCR	>15 WHCR (≤15 km)
**OWNERSHIP**				
TOTAL CONS.	50.6	70.9	56.6	81.1
USFWS	*38.6*	*52.5*	*45.3*	*67.9*
OTHER FEDERAL	*1.3*	*3.2*	*1.9*	*1.9*
STATE AGENCY	*7.0*	*10.8*	*7.5*	*7.5*
CONS. NGO	*3.8*	*4.4*	*1.9*	*3.8*
PRIVATE	49.4	29.1	43.4	18.9

**LOCATIONS**				
QUIVIRA NWR, KS - USFWS	21.5	22.2	20.8	20.8
SALT PLAINS NWR, OK - USFWS	15.2	23.4	20.8	39.6
CHEYENNE BOTTOMS, KS - KDPWT	5.7	5.7	3.8	3.8
PLATTE RIVER, NE – Multiple ORGs	3.8	3.8	3.8	3.8
OTHER (<2.5% per site)	53.8	44.9	50.8	32.0

**DESIGNATION**				
Critical Habitat	44.3	55.1	49.1	67.9
OTHER	55.7	44.9	50.9	32.1

Notes: N = 158 observations of Whooping Crane groups >10 individuals and 53 observations of Whooping Crane groups >15 individuals. “CONS. NGO” = conservation non-governmental organization. “KDPWT” = Kansas Department of Wildlife, Parks and Tourism. “ORGs” = organizations.

## 4 Discussion

Our results demonstrate that Whooping Crane group size (and variation in group size) increased over time as well as in relation to Whooping Crane population via several metrics. However, statistical models with “observation year” as the independent variable outperformed corresponding models with “Whooping Crane population” as the independent variable in all cases, suggesting that factors aside from population growth are contributing to increases in Whooping Crane migratory group size. As population size has increased variability in Whooping Crane migratory behavior (i.e. – flocking) has also increased, suggesting a reasonable level of species’ resilience and adaptability to landscape-level changes such as wetland loss (Wolf and Weissing, 2012; Pearse et al., 2018a). Models assessing the frequency of large Whooping Crane groups (x ≥ 5 and x ≥ 10) by observation year demonstrated the best fits of the data, indicating that the strongest trend across years is the increasing frequency of “large” groups. These results are supported by our finding Whooping Crane groups of 7–9 and ≥10 have increased as a proportion of total groups detected while the percent of observations including individual Whooping Cranes has decreased significantly. The marked increase in groups of ≥10 since the 1990s and the relatively steady increase in groups of 7–9 may suggest that both the frequency and magnitude of large Whooping Crane groups may continue to increase compared to historic record (Austin and Richert, 2001; Sharpe et al., 2001). It is likely that conspecific attraction is a contributing factor to this trend, however, is also likely that these large gatherings are an indicator of limited wetland availability in some locations.

Freshwater emergent wetlands have declined more than any other wetland type in the United States since the 1950s and these losses have been particularly pronounced in the Great Plains as a result of drainage for agricultural development (Dahl, 2000; Tang et al., 2012). As Stahlecker (1992, 1997) notes, the availability of wetland habitat for Whooping Cranes is far more limited in the southern Great Plains, particularly Oklahoma, in comparison to the central and northern Great Plains. Johnson et al. (2012) found that 60% of playa wetlands have been lost from the southern Great Plains through sedimentation and development and that 95.3% of those remaining are in reduced physical condition. Stahlecker (1992) found that there were only between two and four suitable Whooping Crane roost locations available per 100 km^2^ of migration corridor in Oklahoma. Tang et al. (2012) determined that 85% of the playa wetlands in the Rainwater Basin region of Nebraska, south of the Platte River, have been lost and the majority of them have limited restoration potential. Our results indicate a majority of Whooping Crane groups of 15 or greater (60.4%) were observed within 15 km of Quivira NWR and Salt Plains NWR, suggesting limited stopover habitat availability has likely contributed to the increasing frequency of large groups observed in recent decades.

Decreases in available wetland habitat throughout the migration corridor may increase flocking simply due to a lack of available habitat for wider dispersal (Skagen and Knopf, 1994; Sundar, 2006). This phenomenon may be intensified where significant tracts of quality habitat exist within an expanse of low quality habitat (Skagen and Knopf, 1994). Given the small percentage of protected land in the Great Plains (Cordell et al., 2013; Vincent et al., 2017), it is notable that a high proportion of sites supporting large groups are managed by conservation organizations, particularly federal land management agencies. Nearly half of all groups 15 or larger were detected at sites designated as critical habitat for the Whooping Crane under the Endangered Species Act (USFWS, 1978). Relatedly, Pearse et al. (2015) found that grid cells with a higher density of Whooping Crane stopovers contained a higher percentage of conservation protected lands. Pearse et al. (2015) found that 27% of core Whooping Crane use areas had some level of conservation protection compared to just 10% of the migration corridor at large. However, these estimates of protected lands included privately owned parcels enrolled in conservation programs with both temporary and permanent easements in addition to state and federal lands. Given the temporary nature of some of these land protections this estimate of conservation landcover within the migration corridor may be high (Pearse et al., 2015).

The combined ownership of federal land management agencies (USFWS, U.S. National Park Service, U.S. Bureau of Land Management, U.S. Forest Service, etc.) constitutes a small percentage (2.2%) of the states within the 95% Whooping Crane migration corridor (Vincent et al., 2017; range = 0.5% (KS) – 5.4% (SD), Appendix 3). However, 39.9% of Whooping Crane groups of 10 individuals or larger and 47.2% of groups 15 or larger were observed on lands protected by federal land management agencies, particularly the USFWS. Furthermore, an even higher percentage of large groups were detected in close proximity (≥15 km) to federal lands, suggesting Whooping Cranes potentially used these sites before or after being observed. Federally protected lands are scattered across the Great Plains states, while the 50% migration corridor is centered within these states and accounts for about 9.4% of their total land area (Cordell et al., 2013, Pearse et al., 2018b; range = 4.9% (TX) – 20.9% (ND), Appendix 3). Our findings, in addition to this spatial data, suggest that a relatively small percentage of the 50% migration corridor is within 15 km of conservation lands managed by the federal government, but that these habitats may be increasingly valuable to migrating Whooping Cranes as indicated by the frequency of large groups detected at these sites.

Federally protected wetlands have likely maintained quality wetland habitats in the Great Plains where they would otherwise have disappeared (Shaw and Fredine, 1971; Dahl, 2000). These efforts have built a level of resilience into the Great Plains ecosystem and helped recover and maintain robust waterbird migrations (Shaw and Fredine, 1971; Skagen and Knopf, 1994; Webb et al., 2010). Our results suggest protected wetlands within the Great Plains, particularly those conserved by the USFWS and those designated as critical habitat for the Whooping Crane, are providing important stopover areas where wetland habitat is otherwise sparse. It is likely that large Whooping Crane groups will increasing occur within these protected wetlands unless habitat restoration efforts are significantly increased, and this trend could be exacerbated by climate change. The arid climatic conditions of the western United States have been expanding eastward with climate change, which is expected to negatively impact basin wetlands' (i.e. - playa lakes, etc.) ecological function, habitat availability, and therefore waterbirds in the southern Great Plains (Covich et al., 1997; Chavez-Ramirez and Wehtje, 2012; Reese and Skagen, 2017; Seager et al., 2018). Research suggests we are already observing the cumulative effects of wetland habitat loss and climate change on North American crane species. Pearse et al. (2018a) demonstrated that the AWBP's migration corridor is shifting east at a rate of 1.2 km/year and research suggests droughts in the southern Great Plains have been a major driver of irregular Whooping Crane and Sandhill Crane wintering distributions in recent years (Wright et al., 2014; Harner et al., 2015; Caven et al., 2019b).

Nonetheless, large group stopovers were also relatively common in Nebraska within the Platte and Loup River systems in the spring as well as in prairie potholes wetlands in North Dakota during the fall. As Stahlecker (1997) notes Whooping Crane habitat is available throughout the migration corridor in Nebraska, yet we still observed a number of large Whooping Crane groups in this area. However, with significantly less frequency than at large playa-basin wetland complexes in the southern Great Plains. The fact that 69% of large groups were observed within the 50% migration corridor suggests that conspecific attraction may play a role in the formation of large Whooping Crane groups as well. The relatively restricted geographic distribution of large group observations becomes even more apparent considering the 50% use area only represents 23% of the overall geographic area within the migration corridor delineated by Pearse et al. (2018a).

Alonso et al. (1994) suggests that White Storks (*Ciconia ciconia*) perceive patch quality as dependent on the number of birds already foraging. Relatedly, Sundar (2006) found that a number of large waterbird species were good indicators of habitat health and that flocking was favored in patchy environments. Interestingly, Alonso et al. (2004) found that a minority (2%) of Common Crane (*Grus grus*) pairs wintering in Spain were territorial, exclusively when they were supporting young from the previous breeding season and appropriate habitat with defensible resources existed. Additional research demonstrates similarly context-specific variation in territoriality regarding Whooping Cranes (Chavez-Ramirez, 1996; Thompson, 2018). Chavez-Ramirez (1996) demonstrated that although Whooping Cranes generally establish wintering territories within salt marsh habitats in south Texas, they were more gregarious in upland foraging habitats. Concurrently, Thompson (2018) found that Whooping Cranes of the reintroduced Eastern Migratory Population (EMP) that wintered further north (Illinois, Indiana, Kentucky) spent more time in groups, had larger home ranges, and foraged more frequently in agricultural habitats than those cranes wintering in the central (Tennessee, Alabama) or southern (Georgia, Florida, Louisiana) portion of the EMP wintering range.

As Alonso et al. (2004) notes territorial behavior can be expected when food sources are predictably distributed and economically defendable, while group foraging is favored when patches of food are ephemeral, irregularly distributed, yet locally abundant. Additionally, territoriality varies across life stage and annual cycle, as Whooping Cranes generally do not exhibit territorial behavior as subadults or during migration broadly (Urbanek and Lewis, 2015). Just as Whooping Cranes vary in territoriality by context including habitat, forage distribution, life stage, and annual cycle, they likely also vary in gregariousness or sociality (Chavez-Ramirez, 1996; Alonso et al., 2004; Thompson, 2018). Various migration stopover sites and/or habitats within may provide a context for Whooping Cranes in which the benefits of gathering in larger flocks substantially outweigh those of persisting in traditional family groups, pairs, or as individuals. Conspecific attraction may be highest at stopover sites where resources are distributed unpredictably and other Whooping Cranes could indicate forage availability or those with potential predation threats where collective vigilance could offer additional protection and the opportunity to increase foraging rates (Alonso et al. 1994, 2004; Muller et al., 1997; Sundar, 2006).

Grouping behavior may also be driven by different factors at different locations, particularly as migration chronology varies by flock demographics, latitude, and season (Kuyt, 1992; Johns, 1992). Unpaired and non-breeding subadults likely face fewer time constraints on their northward migration and may occasionally stage for a period of weeks in quality habitats such as riverine wetlands in Nebraska, which as a state has the second highest total occurrence of large group observations (≥10 individuals) in the spring after Kansas (Lingle et al., 1991; PRRIP, 2017). Subadult Whooping Cranes are often the first to depart the breeding grounds and regularly stage in south-central Saskatchewan, foraging in agricultural fields and wetlands, occasionally with flocks of Sandhill Cranes (Johns, 1992; Johns et al., 1997). The “southern prairies” of Saskatchewan are an important stopover location, particularly during the fall, where large flocks of Whooping Cranes have been observed (Armbruster, 1990; Johns, 1992; Johns et al., 1997, FOTWW 2018). In the fall family groups can take an extended period of time to complete their southerly migration as colts are unaccustomed to the difficulty of long duration flights (Lingle et al., 1991; Kuyt, 1992; Richert, 1999). Any process that prolongs migration and the number of stopover sites used would theoretically increase the exposure of Whooping Crane flocks to each other.

Another potential driver of large group occurrences may be locations that serve as staging grounds as opposed to typical stopover locations. However, these two terms are often poorly differentiated in the ornithological literature (Warnock, 2010). Warnock (2010) suggests that staging sites are set apart from stopover sights by predictable abundant foraging resources, which prepare individuals for challenging portions of their migration, such as long flights over areas of poor habitat. Staging sites usually differ from stopover sites as individual birds tend to stay significantly longer and gain substantially more body mass (Ma et al., 2013). Pearse et al. (2015) found differential site use intensity throughout the migration corridor noting that “extended-use core intensity” sites made up only 13% of stopover sites but accounted for 42% of Whooping Crane stopover days. Melvin and Temple (1982) and Johns (1992) suggest that staging areas generally occur in the first 25% of the migration corridor regarding North American crane species. Johns (1992) notes that the “southern plains” of south-central Saskatchewan are about 20–25% into the migration route south. Salt Plains NWR is about 23% and Quivira NWR is about 27% of the way north on the spring migration route to the breeding grounds (Google, 2018).

Wetland loss has likely resulted in increasing concentrations of Whooping Cranes in aquatic habitats, which pose significant disease risks including avian cholera, botulism, tuberculosis, and coccidiosis (Windingstad, 1988; Blanchong et al., 2006; CWS and USFWS, 2007). A number of other studies have documented potentially harmful parasites in Whooping Cranes that can result from high waterbird densities, including from Sandhill Cranes (*Grus canadensis*), with which Whooping Cranes often forage during migration (Lu et al., 2013; Vogel et al., 2013; Bertram et al., 2017). Moreover, increasingly large groupings of Whooping Cranes present a potential mass mortality risk from extreme weather events like tornadoes and hail storms (Lingle, 1997; Higgins and Johnson, 1978; Narwade et al., 2014). For instance, Narwade et al. (2014) documented over 60,000 avian mortalities across 26 sites following a widespread hailstorm in Maharashtra, India. Lingle (1997) documented several thousand dead Sandhill Cranes following a late March blizzard in the Central Platte River Valley in 1996. Efforts to protect and restore wetland habitat, particularly in the southern Great Plains of the United States where stopover options may be particularly limited, could help mitigate the disease and weather related risks Whooping Cranes face during their migration (Higgins and Johnson, 1978; Meine and Archibald 1996; CWS and USFWS, 2007).

It is important to note that the Whooping Crane tracking partnership data used here is derived from public sightings and include an unquantified amount of observational bias as detection effort is not equally distributed across the landscape (Tacha et al., 2010), with potentially more observational effort dedicated to areas managed by professional conservation organizations or near human populations. For example, the total percentage of the large flock stopovers occurring at locations managed by professional biologists may be inflated in our calculations via increased observational effort and expertise in such locations (Tacha et al., 2010). However, there is no logical reason for systematic bias in the group size estimates provided between locations where Whooping Cranes are detected or across survey years via the public sightings database. The data support this assertion as several locations have regularly reported Whooping Cranes during this study, but only a subset of those locations regularly reported large Whooping Crane groups. While large groups may be more easily detected than small groups, this bias should arguably be consistent across sites and years in the database. Despite the limitations of our dataset, it is clear that occurrences of large flocks are disproportionally occurring in large, diverse, and reliable basin wetlands in the southern Great Plains, near the center of the migration corridor, and at sites that serve as staging areas. It is likely that population growth, habitat availability, and conspecific attraction all play a role in the formation of large Whooping Crane groups, along with additional factors such as migration phase and season.

## 5 Conclusions

We provide evidence to suggest that large Whooping Crane flocks, or groupings of flocks, are more likely to occur near the center of the migration corridor. Our results also suggest that large groups may form where high quality reliable (perennial ponding) wetland habitat is available in parts of the migration corridor where habitat and therefore quality alternatives are limited. It may also be true that large group stopovers occur in areas associated with extended stays, namely staging areas in the southern Great Plains of the United States, and from other published works, the southern prairies of Saskatchewan, Canada. Further research should investigate whether stopover habitat quality, complexity, extent, or availably, at various spatial scales, plays a role in the formation of large migratory gatherings of Whooping Cranes.

Large gatherings of Whooping Cranes present several risks to the population, including from disease associated with the high concentrations of other waterbirds at some important stopover locations (i.e. – Lesser Snow Geese (*Chen caerulescens caerulescens*)). However, these gatherings may also represent instinctual and adaptive behavior historically exhibited during migration that provided cues to quality stopover habitats and safety in unfamiliar locales, which disappeared as Whooping Crane populations hovered at near extinction levels over the last century. Our data indicate that Whooping Crane groups of 10 or larger are increasing at a rate exceeding population growth, suggesting that large gatherings are likely to become increasingly common in particular locations. Wetland restoration and protection efforts, particularly in the southern Great Plains where habitat might be limited, may help disperse Whooping Cranes and mitigate the risk posed by the largest aggregations. However, it is likely that we will continue to observe increasing concentrations of Whooping Cranes, and conservationists will need to adapt policies and management actions accordingly.

## Declarations

### Author contribution statement

Andrew J. Caven, Matt Rabbe: Conceived and designed the experiments; Performed the experiments; Analyzed and interpreted the data; Contributed reagents, materials, analysis tools or data; Wrote the paper.

Jenna Malzahn: Analyzed and interpreted the data; Wrote the paper.

Anne E. Lacy: Contributed reagents, materials, analysis tools or data; Wrote the paper.

### Funding statement

This work was supported by the U.S. Fish and Wildlife Service as well as the Platte River Whooping Crane Maintenance Trust.

### Competing interest statement

The authors declare no conflict of interest.

### Additional information

Data associated with this manuscript can be obtained by written request to the U.S. Fish and Wildlife Service, Nebraska Ecological Services Field Office, 9325 S. Alda Rd, Wood River, Nebraska, 68883, USA, electronically by request at nebraskaes@fws.gov, or by phone at (308)-382-6864. Users are required to review and agree to the terms and conditions of a data use agreement provided by the U.S. Fish and Wildlife Service prior to obtaining locational data associated with this sensitive endangered species.

Supplementary content related to this article has been published online at https://doi.org/10.1016/j.heliyon.2020.e03549.
