# Supplementary material for: Trends in the occurrence of large Whooping Crane groups during migration in the great plains, USA
Source: Heliyon. 2020 Apr 2;6(4):e03549. doi: 10.1016/j.heliyon.2020.e03549 (PMC7132073; doi:10.1016/j.heliyon.2020.e03549)
Supplement: Appendix 1 [file mmc1.docx]

APPENDIX 1. Variable description, reference number (corresponding to Materials and Methods text), and dependent variable name (corresponding to Table 1 in Results) for dependent variables used to assess the trends in the size of migrating groups of AWBP Whooping Cranes (WHCRs) from 1942 to 2018.

| **Dependent Variable Name** | **Reference No.** | **Variable Description** |
| --- | --- | --- |
| No. per Group | 1 | Number of individual WHCRs observed per group |
| No. per Group – Spr. | 2 | Number of individual WHCRs observed per group during spring migration |
| No. per Group – Fall | 3 | Number of individual WHCRs observed per group during fall migration |
| Juveniles per Group | 4 | Number of juvenile WHCRs observed per group |
| Adults per Group | 5 | Number of adult WHCRs observed per group |
| Max in Group per Yr. | 6 | Maximum number of WHCRs observed in one group each year |
| Max in Group per Spr. | 7 | Maximum number of WHCRs observed in one group each spring migration |
| Max in Group per Fall | 8 | Maximum number of WHCRs observed in one group each fall migration |
| Mean in Group – Yr. | 15 | Mean number of WHCRs observed per group each survey year |
| Mean in Group – Spr. | 16 | Mean number of WHCRs observed per group each spring migration |
| Mean in Group – Fall | 17 | Mean number of WHCRs observed per group each fall migration |
| Count of Groups >5 – Yr. | 9 | Number of Whooping Crane groups larger than five individuals per year |
| Count of Groups >5 – Spr. | 11 | Number of Whooping Crane groups larger than five individuals spring migration |
| Count of Groups >5 – Fall | 13 | Number of Whooping Crane groups larger than five individuals per fall migration |
| Count of Groups >10 – Yr. | 10 | Number of Whooping Crane groups larger than ten individuals per year |
| Count of Groups >10 – Spr. | 12 | Number of Whooping Crane groups larger than ten individuals per spring migration |
| Count of Groups >10 – Fall | 14 | Number of Whooping Crane groups larger than ten individuals per fall migration |
