# Supplementary material for: Trends in the occurrence of large Whooping Crane groups during migration in the great plains, USA
Source: Heliyon. 2020 Apr 2;6(4):e03549. doi: 10.1016/j.heliyon.2020.e03549 (PMC7132073; doi:10.1016/j.heliyon.2020.e03549)
Supplement: Appendix 2 [file mmc2.docx]

APPENDIX 2. Comparison of Whooping Crane group sizes observed across states within the 95% migration corridor (Pearse et al. 2018a) in both the spring and fall using a one-way ANOVA with a Tukey Honest Significant Difference Post-hoc Test.

| **STATE-STATE** | **Diff.** | **Lwr CI** | **Upr CI** | ***p*-adj** |
| --- | --- | --- | --- | --- |
| **SPRING** |  |  |  |  |
| NEBRASKA-KANSAS | -1.5004 | -2.4492 | -0.5516 | 0.0000*** |
| NORTH DAKOTA-KANSAS | -1.6199 | -2.6501 | -0.5897 | 0.0000*** |
| OKLAHOMA-NORTH DAKOTA | 2.3295 | 0.7113 | 3.9477 | 0.0002*** |
| OKLAHOMA-NEBRASKA | 2.2100 | 0.6424 | 3.7776 | 0.0003*** |
| SOUTH DAKOTA-OKLAHOMA | -2.1626 | -3.9256 | -0.3995 | 0.0039** |
| SOUTH DAKOTA-KANSAS | -1.4530 | -2.6984 | -0.2075 | 0.0081** |
| OKLAHOMA-MONTANA | 2.5352 | -0.2469 | 5.3173 | 0.1123 |
| MONTANA-KANSAS | -1.8256 | -4.3122 | 0.6609 | 0.3886 |
| TEXAS-OKLAHOMA | -1.4189 | -3.8575 | 1.0198 | 0.7330 |
| TEXAS-NORTH DAKOTA | 0.9106 | -1.1314 | 2.9526 | 0.9387 |
| OKLAHOMA-KANSAS | 0.7096 | -0.9752 | 2.3943 | 0.9580 |
| TEXAS-NEBRASKA | 0.7911 | -1.2111 | 2.7933 | 0.9730 |
| TEXAS-MONTANA | 1.1164 | -1.9318 | 4.1646 | 0.9845 |
| TEXAS-SOUTH DAKOTA | 0.7437 | -1.4149 | 2.9023 | 0.9903 |
| TEXAS-KANSAS | -0.7093 | -2.8045 | 1.3859 | 0.9915 |
| SOUTH DAKOTA-MONTANA | 0.3727 | -2.1676 | 2.9129 | 1.0000 |
| NORTH DAKOTA-NEBRASKA | -0.1195 | -0.9443 | 0.7053 | 1.0000 |
| SOUTH DAKOTA-NORTH DAKOTA | 0.1669 | -0.9869 | 1.3207 | 1.0000 |
| NEBRASKA-MONTANA | 0.3252 | -2.0835 | 2.7340 | 1.0000 |
| NORTH DAKOTA-MONTANA | 0.2058 | -2.2362 | 2.6477 | 1.0000 |
| SOUTH DAKOTA-NEBRASKA | 0.0474 | -1.0343 | 1.1292 | 1.0000 |
| **FALL** |  |  |  |  |
| OKLAHOMA-NORTH DAKOTA | 1.4981 | 0.6245 | 2.3717 | 0.0000*** |
| TEXAS-OKLAHOMA | -2.0620 | -3.2718 | -0.8521 | 0.0000*** |
| OKLAHOMA-NEBRASKA | 1.2645 | 0.3258 | 2.2031 | 0.0006*** |
| SOUTH DAKOTA-OKLAHOMA | -1.4269 | -2.5462 | -0.3076 | 0.0017** |
| TEXAS-KANSAS | -1.2980 | -2.4320 | -0.1639 | 0.0097** |
| NORTH DAKOTA-KANSAS | -0.7341 | -1.4993 | 0.0310 | 0.0749 |
| OKLAHOMA-KANSAS | 0.7640 | -0.0520 | 1.5800 | 0.0932 |
| TEXAS-NEBRASKA | -0.7975 | -2.0227 | 0.4278 | 0.6216 |
| SOUTH DAKOTA-KANSAS | -0.6629 | -1.6998 | 0.3740 | 0.6494 |
| OKLAHOMA-MONTANA | 1.8653 | -1.0732 | 4.8038 | 0.6602 |
| NEBRASKA-KANSAS | -0.5005 | -1.3392 | 0.3382 | 0.7465 |
| TEXAS-NORTH DAKOTA | -0.5638 | -1.7400 | 0.6123 | 0.9332 |
| TEXAS-SOUTH DAKOTA | -0.6350 | -2.0036 | 0.7335 | 0.9471 |
| MONTANA-KANSAS | -1.1013 | -4.0094 | 1.8068 | 0.9897 |
| NORTH DAKOTA-NEBRASKA | -0.2336 | -1.1284 | 0.6612 | 0.9997 |
| NEBRASKA-MONTANA | 0.6008 | -2.3441 | 3.5457 | 1.0000 |
| SOUTH DAKOTA-MONTANA | 0.4384 | -2.5690 | 3.4457 | 1.0000 |
| SOUTH DAKOTA-NEBRASKA | -0.1624 | -1.2984 | 0.9735 | 1.0000 |
| NORTH DAKOTA-MONTANA | 0.3672 | -2.5576 | 3.2920 | 1.0000 |
| TEXAS-MONTANA | -0.1967 | -3.2389 | 2.8455 | 1.0000 |
| SOUTH DAKOTA-NORTH DAKOTA | 0.0712 | -1.0116 | 1.1540 | 1.0000 |
