# Supplementary material for: Trends in the occurrence of large Whooping Crane groups during migration in the great plains, USA
Source: Heliyon. 2020 Apr 2;6(4):e03549. doi: 10.1016/j.heliyon.2020.e03549 (PMC7132073; doi:10.1016/j.heliyon.2020.e03549)
Supplement: Appendix 3 [file mmc3.docx]

APPENDIX 3. Percentage and area of each state within the 95% migration corridor (Pearse et al. 2018) owned by federal land management agencies and within the 50% migration corridor. Data for area owned by federal land management agencies from Vincent et al. (2017) and data from area within the 50% migration period from Pearse et al. (2018b).

| **State** | **Federal Land** | | **Migration Corridor** | | **Total Land** |
| --- | --- | --- | --- | --- | --- |
|  | *Hectares* | *%* | *Hectares* | *%* | *Hectares* |
| **Texas** | 1,210,396 | 1.8 | 3,349,435 | 4.9 | 68,075,308 |
| **Oklahoma** | 283,683 | 1.6 | 1,334,569 | 7.5 | 17,841,667 |
| **Kansas** | 110,474 | 0.5 | 1,042,013 | 4.9 | 21,250,353 |
| **Nebraska** | 221,354 | 1.1 | 2,552,411 | 12.9 | 19,842,434 |
| **South Dakota** | 1,072,182 | 5.4 | 3,377,709 | 17.1 | 19,781,829 |
| **North Dakota** | 703,405 | 3.9 | 3,765,654 | 20.9 | 17,989,296 |
| Totals | 3,601,494 | 2.2 | 15,421,791 | 9.4 | 164,780,887 |
